# Results of a randomised controlled study on the efficacy of a combination of saline irrigation and Sinupret syrup phytopreparation in the treatment of acute viral rhinosinusitis in children aged 6 to 11 years

**DOI:** 10.1186/s40816-018-0082-y

**Published:** 2018-09-06

**Authors:** Vasyl I. Popovich, Halyna V. Beketova

**Affiliations:** 1 Department of Otorhinolaryngology, Ivano-Frankivsk University, 76000 Galitskaya str. 2, Ivano-Frankivsk, Ukraine; 2 Department of Paediatrics, Kiev National Medical Academy of Postgraduate Education, Dorogojitskaya str. 9, Kiev, 02000 Ukraine

**Keywords:** Rhinosinusitis, Herbal medicine, Sinupret® syrup, Rhinorrhoea

## Abstract

**Background:**

This study was designed to demonstrate the effectiveness of the complex herbal medicine Sinupret® syrup in the treatment of acute viral rhinosinusitis in children.

**Methods:**

Patients aged 6 to 11 years were included in a randomised controlled study with two parallel groups. Both groups received standard treatment including Weber’s douche and symptomatic medicine on therapeutic grounds. Isotonic sea salt solution was applied four times daily for 10 days. The intervention group received Sinupret® syrup three times daily as add-on therapy. Using a five-point scale (0–4 points) the physicians evaluated the following symptomatic parameters within four successive visits (Day 0, 5, 7, and 10): nasal congestion, nasal discharge, post-nasal drip, headache, and facial pain. Using a 11 -point scale (0–10 points), each patient gave a daily self-assessment of the following parameters from Day 1 to Day 10: rhinorrhoea, headache, and facial pain.

**Results:**

In total, 184 patients (mean age 8.1 years) were included. In accordance with physicians’ assessment compared to saline irrigation alone, significant improvements were detected in three out of five symptomatic parameters under combined treatment including Sinupret® syrup as assessed by the physicians. The between-group differences in the severity of facial pain and headache were insignificant. The Sinupret® group also showed a trend to an antibiotic-sparing effect (2.17% in the Sinupret® group vs. 5.26% in the control group). Further, the frequency of the transition of viral rhinosinusitis to the post-viral phase tended to decrease (1.08% in the Sinupret® group vs. 5.26% in the control group). No adverse reactions to the herbal medicine occurred during the study period.

**Conclusion:**

The complex herbal medicine Sinupret® syrup alleviates effectively the symptoms of acute viral rhinosinusitis in children. Furthermore, the prescription of antibiotics was also reduced.

## Background

Acute rhinosinusitis (ARS) is the most common infectious disease and apart from personal discomfort with reduced quality of life, it has a vast social and economic impact [1]. The concept of “rhinosinusitis” was introduced in the recent years as inflammation had been proved to occur simultaneously in the nose and paranasal sinuses. Each case of common cold with rhinitis symptoms should be considered as ARS. According to the European Position Paper on Rhinosinusitis and Nasal Polyps (EPOS 2012), ARS is characterised, apart from inflammation of nasal passages and paranasal sinuses, by two or more specific symptoms: nasal congestion or nasal discharge; besides facial pain/pressure, or anosmia/hyposmia may occur [2]. Additional symptoms such as fever, asthenia, or headache may be observed.

As a rule, ARS is a self-limiting disease lasting for 7 to 14 days. ARS includes a viral phase (acute viral rhinosinusitis) and a post-viral phase. In EPOS 2007, the term “viral ARS” was chosen to indicate that most of the ARS cases are not bacterial. Only about 5% of ARS may be diagnosed as acute bacterial rhinosinusitis (ABRS). Typical ABRS symptoms include nasal discharge, unilateral facial pain, or tenderness on palpation in the area of sinus projection (in children older than 9–12 years), toothache, subsequent exacerbation after primary amelioration, hyperthermia, and neutrocytosis.

Acute rhinosinusitis is the fifth frequent diagnosis for antibiotic prescription, although there is no evidence that antibacterial therapy reduces the duration of the disease. Frequent and groundless use of antibiotics causes increased resistance resulting in the need for alternative therapeutic strategies based on the scientific data. The main cause of ARS in the first 10 days of the disease is basically a number of viruses (rhinovirus, parainfluenza virus type 1 and type 2, coronavirus, influenza virus), all of which increase the concentration of proinflammatory cytokines and neutrophils [3]. In addition, their activity leads to disorders of mucociliary clearance due to damage of ciliated cells, as well as to increase of thick secretion. These changes lead to the gradual deterioration in the quality of the ostiomeatal complex, disorder of ventilation and drainage of paranasal sinuses. The similar type of reaction is also observed in the context of bacterial infection. Thus, ARS may be easily misdiagnosed as a bacterial infection and therefore groundlessly treated with antibiotics, which do not contribute to recovery at this stage of the disease.

A common strategy for the treatment of acute viral rhinosinusitis is to reduce symptom severity, to minimise disease duration, to prevent transformation into post-viral and bacterial rhinosinusitis, as well as to prevent further progression into a chronic disease. The use of antibiotics, nasal decongestants, antihistamines, homeopathic medicines, and mucolytic agents in acute viral rhinosinusitis is groundless as their benefit has not yet been proved. According to EPOS 2012, pharmaceutical symptomatic treatment for acute viral rhinosinusitis includes: therapeutic irrigation with isotonic sea salt solution and non-steroidal anti-inflammatory drugs or antipyretics (NSAIDs, aspirin or paracetamol). An alternative strategy is the use of herbal medicines, which are able to suppress a number of pathological processes [4–6].

One example is the complex herbal medicine Sinupret®, which includes gentian, primula, elder, verbena, and sorrel. It has been proven that this herbal medicine intensifies ciliary activity in vitro [7] and it shows anti-inflammatory activity in experiments on animals [8]. It has a wide range of pharmacologic properties including mucolytic, secretomotor, antiviral, anti-inflammatory, and immunomodulatory action. Jund et al. [9] conducted a randomised double-blind, placebo-controlled study with 386 adult patients with acute viral rhinosinusitis. The active treatment group received a daily dose of 3 × 160 mg of the phytopreparation within 15 days. The active treatment group showed a significant improvement compared to the placebo group based on the results of a sinonasal test including total index, nasal symptoms, rhinogenous symptoms and overall quality of life.

A study on the efficacy of Sinupret® syrup in the treatment of viral ARS in school-aged children (6 to 11 years old) has not been conducted previously. Here, we report the results of a randomised controlled study in children aged 6 to 11 years applying Sinupret® as a syrup. Our study is similar to the trial conducted in adults by Jund et al.

## Methods

### Study plan

This study was a prospective, multicentre, interventional, randomised study of viral ARS treatment in children aged 6 to 11 years. This study compared complex phytotherapeutic treatment with Sinupret® syrup in combination with therapeutic irrigation to standard therapeutic irrigation (Table 1).

**Table 1:** Study treatments

Groups	Pharmaceutical drug	Dosage	Duration
Sinupret®	Therapeutic irrigation (isotonic sea salt solution)	4 times daily	10 days
	Phytopreparation, syrup (Sinupret®)	(3.5 mL), 3 times daily	
	Symptomatic medications (paracetamol, decongestants) by indications	Age-specific dosage	
Control	Therapeutic irrigation (isotonic sea salt solution)	4 times daily	10 days
	Symptomatic medications (paracetamol, decongestants) by indications	Age-specific dosage	

Sinupret® is a herbal medicine widely used for the treatment of different respiratory tract diseases including rhinosinusitis. The composition is a mixture derived from parts of four plants: gentian root (Radix Gentianae), primrose flowers with calyx (Flores Primulae cum Calibus), flowers of elderberry (Flores Sambuci), European vervain herb (Herba Verbenae), and sorrel grass (Herba Rumісіs) (1:3:3:3:3).

Both groups received symptomatic medications (paracetamol or nasal decongestants if necessary), and both groups were applied therapeutic irrigation with isotonic sea salt solution four times daily. The Sinupret® group additionally received the herbal medicine Sinupret® syrup three times daily in an age-specific dosage of 3.5 mL.

### Study population

The study population consisted of 184 children, 98 boys and 86 girls. Using the method of opaque sealed and sequentially numbered envelopes, 96 children were distributed to the Sinupret® group, and 88 children to the control group.

### Inclusion criteria

The main inclusion criteria were acute viral rhinosinusitis with acute symptoms up to 48 h and a total score of sinusitis severity of 8 to 15 points according to major sinusitis severity score (MSS score). For assessment of the MSS score, the five key symptoms were rated by the physicians (0 to 4 points per symptom; summing up to a maximum MSS score of 20 points): nasal discharge, nasal congestion, post-nasal drip, headache, facial pain with (0 — absent, 1 — slight, 2 — moderate, 3 — severe, 4 — very severe).

### Exclusion criteria

- Administration of herbal medicine within 30 days prior to first manifestation of rhinosinusitis.
- Diagnosis of allergic rhinosinusitis.
- Known intolerance to primrose drugs.
- Severe acute disease requiring hospitalisation or treatment with antibiotics.
- Administration of topical corticosteroids.
- Immune deficiency.
- Chronic pathology and anatomical anomalies of ostiomeatal complex, which may influence the outcome of the disease.

### Methodology

During the study period, four visits were conducted: visit 1 (day 0), visit 2 (day 5), visit 3 (day 7), and visit 4 (day 10). Symptoms were assessed by the physicians and the patients. The five key symptoms (nasal discharge, nasal congestion, post-nasal drip, headache, facial pain) were assessed by the physicians (0 to 4 points per symptom) at each visit. In addition, the key complaints: rhinorrhoea, headache, and facial pain were assessed daily by the patients and their parents using a 10–point visual analogue scale.

### Efficacy criteria

The primary criteria were the improvements in symptoms. The secondary criteria were the frequency of transition to antibiotic prescription, exacerbation after day 5 or persistence of symptoms after day 10, and the duration of the disease.

### Data analysis

The data were analysed descriptively. Differences between the two groups were analysed using the paired t-test, using a two-sided 95% confidence interval (95%-CI) with *p < 0.05* indicating statistical significance.

#### Trial registration

This trial was registered in German Clinical Trials Register retrospectively 27. March 2018.

**Trial Acronym**

**ARSiCh DRKS-ID: DRKS00000765**

## Results

### Study population

One hundred sixty-nine patients out of 184 completed the study period of 10 days. In total, 4 patients in the Sinupret® group and 12 patients in the control group were excluded from the study due to protocol violations, and data of these patients were excluded from analysis. Thus, data of 92 patients in the Sinupret® group and 76 patients in the control group were used for analysis.

### Follow-up outcome

By visit 4 (day 10), 1 patient (1.08%) in the Sinupret® group and 4 patients (5.26%) in the control group showed continuation or exacerbation of symptoms after day 5 with no signs of bacterial inflammation. Post-viral rhinosinusitis was diagnosed in these patients. However, these differences were not statistically significant. A similar trend without statistical significance was also observed regarding the terms of recovery (in the Sinupret® group the duration of the disease was 7.85 days vs. 8.88 days in the control group).

### Antibiotics treatment

In total, 2 patients (2.17%) in the Sinupret® group had to take antibiotics compared to 4 patients (5.26%) in the control group. This difference was not statistically significant (*p* > 0.05). In all cases (6 patients) antibiotics administration was initiated at the time of visit 3 due to rise in body temperature (39 °C and higher), sinusitis symptoms exacerbation.

### Symptoms assessed by the physicians

**Fig. 1:**
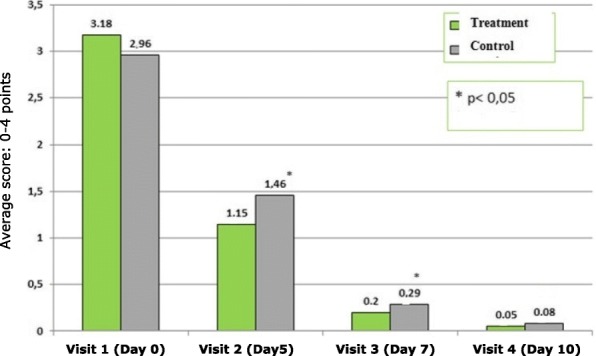
Physicians’ assessment of dynamics of nasal congestion symptoms at visits 1 to 4. Average score (MSS scale: 0–4 points). * *p* < 0.05: Significant difference between groups

Figure 1 shows the physicians’ assessment (by rhinoscopy) of dynamics of nasal congestion symptoms at visits 1 to 4. Both groups showed comparable symptoms at visit 1. At visit 2 and visit 3 nasal congestion was significantly lower in the Sinupret® group than in the control group (*p* = 0.044 and *p* = 0.048 respectively). From visit 3 to visit 4 nasal congestion in both groups showed further symptom reduction and patients were symptom-free at visit 4 .

**Fig. 2:**
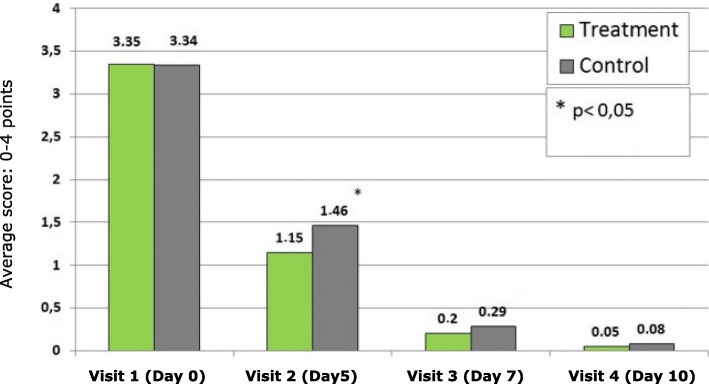
Physicians’ assessment of nasal discharge symptoms at visits 1 to 4. Average score (MSS scale: 0–4 points). * *p* < 0.05: Significant difference between groups

Figure 2 shows the physicians’ assessment of nasal discharge symptoms at visits 1 to 4. Differences of indexes at visit 2 are statistically significant (*p* = 0.037). At visit 3 and visit 4, there is a further decrease of symptom intensity, however, between-group differences did not reach statistical significance anymore.

**Fig. 3:**
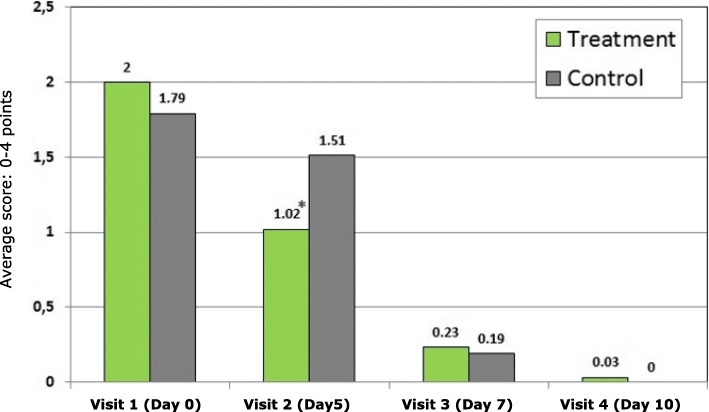
Physicians’ assessment of dynamics of the post-nasal drip symptoms (average score on the MSS scale: 0–4 points). * *p* < 0.05: Significant difference between groups

Figure 3 shows that physicians assessed post-nasal drip symptoms (by pharyngoscopy) to be less severe in the Sinupret® group than in the control group at visit 2 (1.02 vs. 1.51 points, *p* = 0.034). At visit 3 and visit 4 in both groups showed further symptom reduction but without statistical significance.

The physicians’ assessment of headache also showed that this symptom was slightly less severe in the Sinupret® group compared to the control group at visit 2 (0.3 vs. 0.4), but the difference was not significant. No statistically significant difference was observed regarding facial pain between the groups.

**Fig. 4:**
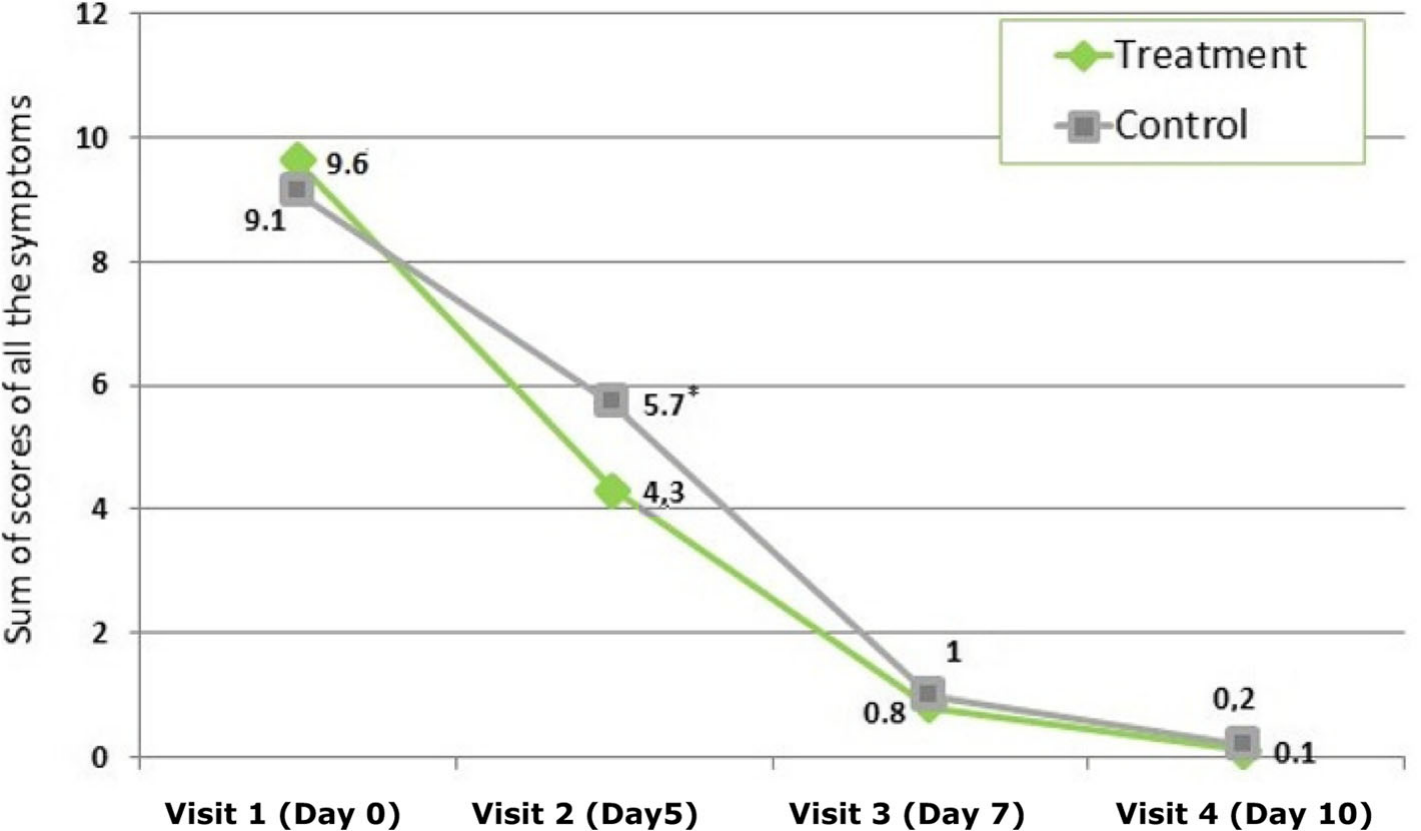
Physicians’ assessment of sinusitis severity for all five symptoms (average summary score, of the MSS scale: 0–20 points). * *p* < 0.05: Significant difference between groups

Figure 4 shows the physicians’ assessment of consolidated figures (in points) of sinusitis severity for all five symptoms. At visit 2 (day 5), a significant difference between the groups (*p* = 0.037) in favour of the treatment group was observed. Symptom reduction persisted over time. However, the between-group comparison becomes statistically insignificant at visit 3 and visit 4.

### Symptoms assessed by the patients

**Fig. 5:**
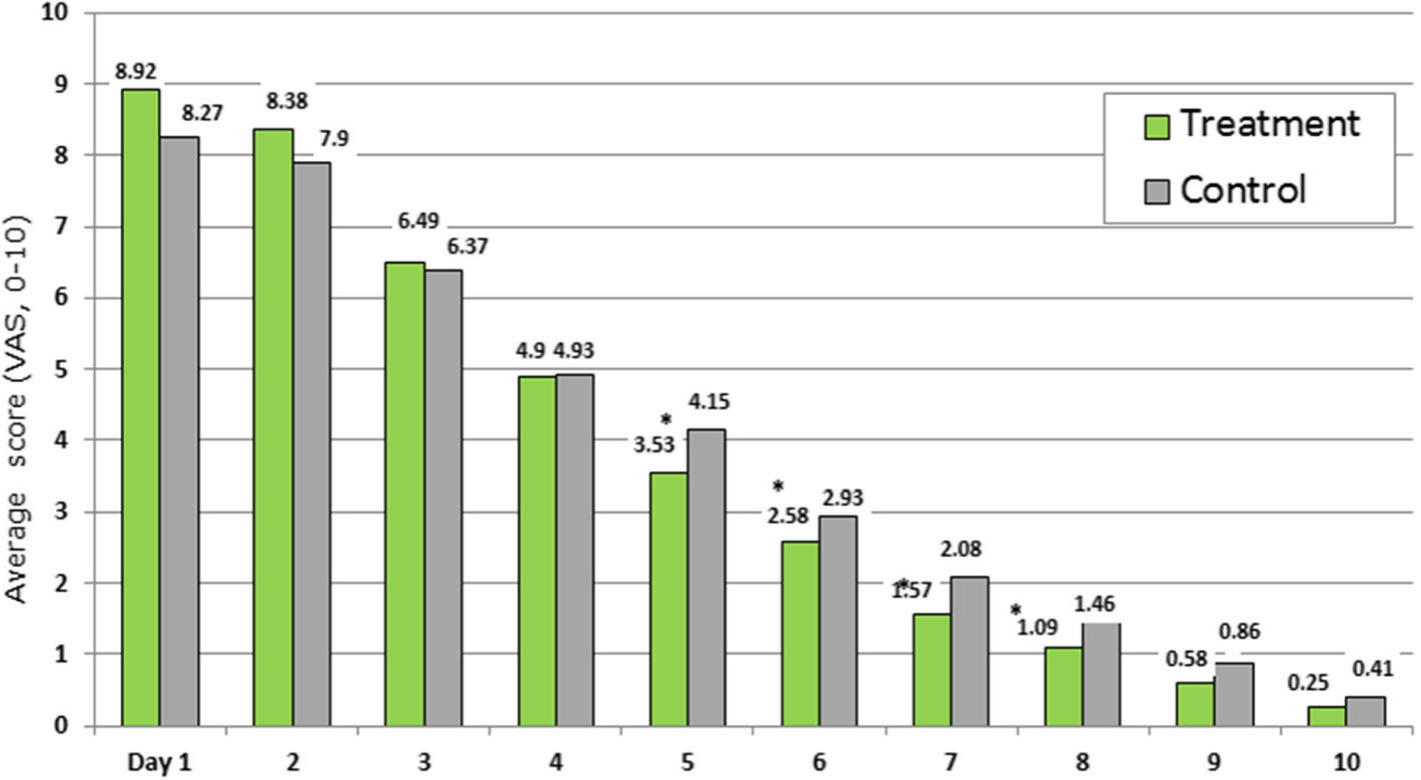
Patients’ self-assessment of their condition during the first 10 days of the treatment for three symptoms (rhinorrhea, facial pain, and headache). * *p* < 0.05: Significant difference between groups

Figure 5 shows patients’ self-assessment (with parental help) of their condition (average score) during the first 10 days of the treatment for three symptoms: rhinorrhoea, facial pain, and headache (0–10 score per symptom). At the beginning of the study (days 1 to 4), patients’ assessment in both groups was similar. A significantly lower level of the main complaints was found in the Sinupret® group on days 5, 6, 7, and 8 (all *p* < 0.05). In general, the patients’ self-assessment of symptom dynamics is consistent with the assessments provided by the physicians at visit 2 (statistically significant difference between groups).

## Discussion

Acute viral rhinosinusitis is a prevalent and economically important disease, involving inflammation of nasal mucosa and paranasal sinuses [1, 2]. Now there is no conventional gold standard for ARS treatment. Antibiotics are not indicated in the treatment of uncomplicated ARS. Saline irrigation proved efficient for improving the symptoms in randomised, placebo-controlled studies [10]. Since 2016, complex herbal preparation Sinupret® has been included in the national clinical guidelines for the treatment of acute rhinosinusitis in Ukraine. There is extensive evidence from in vitro and in vivo studies that the complex herbal medicine Sinupret® possesses a variety of such relevant activities: anti-inflammatory action [8]; stimulation of transepithelial chloride transport, and enhances ciliary beat frequency [7, 11]; antiviral activity [12].

Until now there has been limited knowledge of the beneficial effects of herbal medicines in the treatment of ARS in children. In our previous study, we investigated the effectiveness of Sinupret® in children with acute postviral RS with a positive effect [13]. Our current study result shows that administration of the herbal drug Sinupret® syrup (3.5 ml / 3 times daily) is effective in the treatment of acute viral rhinosinusitis in children aged 6 to 11. Application of this herbal preparation together with saline irrigation and symptomatic treatment gives faster recovery from symptoms, gives a higher rate of complete recovery without the use of antibiotics. In our study, we used irrigation therapy in both groups as a component of the basic therapy with local activity. Impact of irrigation therapy can be assumed as similar in both groups, since the group parameters are comparable. Evaluated differences in severity of symptoms dynamic between both groups can therefore be assumed to the herbal medicine action. An additional herbal medicine with multiple systematic activities could be useful in alleviating the symptoms of acute viral rhinosinusitis and might inhibit the transition to a bacterial infection or postviral rhinosinusitis. Compared to the standard treatment, in the Sinupret group, significant improvements were observed in three out of five key symptoms as assessed by the physician, and nasal congestion as assessed by the patient. In general, assessment of symptoms dynamics by physicians and by patients were consistent. A reduction in the number of patients taking antibiotics and a trend towards the reduction of the disease duration and of the transition of disease to the post-viral rhinosinusitis were also observed. However, the difference in these two parameters is considered not to be statistically significant. During the study in patients with postviral rhinosinusitis the similar results were observed [12]. During the present study, none of the patients showed any adverse reactions to Sinupret® syrup. This fact may be due to a relatively small number of participants however it confirms the good tolerability of the medicine in the form of syrup for use in children.

### Limitations

This was an open-label, randomised, interventional study. Limitations include the absence of virological information and radiologic data. Further, the small sample size limits the accuracy of the results on the disease duration and frequency of antibiotics prescription in the groups.

## Conclusion

Sinupret® is an effective treatment of acute viral rhinosinusitis in children, and it accelerates the relief of the main symptoms. Sinupret® can also help to reduce extensive antibiotics prescription under this condition and reduce the possibility of disease progression to the phase of post-viral rhinosinusitis. This seems to be important in view of the necessity to reduce the undue prescription of antibiotics and the development of bacterial resistance.

## Abbreviations

ABRS: acute bacterial rhinosinusitis
ARS: acute rhinosinusitis
CFTR: cystic fibrosis transmembrane conductance regulator
EPOS: European Position Paper on Rhinosinusitis and Nasal Polyps;
MSS score: major sinusitis severity score
V1, 2, 3, 4: visits 1, 2, 3, 4.
